# Effects of Gamma Irradiation on Biological Yield, Iron and Zinc Accumulation, and Indirect Mineral Bioavailability in Spring Wheat Mutant Lines

**DOI:** 10.3390/biology15191785

**Published:** 2026-10-08

**Authors:** Gulina Doktyrbay, Saule Kenzhebayeva, Saule Atabayeva, Saltanat Asrandina, Nurgul Amangeldi, Rauza Abdukerim, Moldir Turaliyeva, Maira Kazankapova, Xiaodong Liang

**Affiliations:** 1 Department of Biotechnology, Faculty of Biology and Biotechnology, Al-Farabi Kazakh National University, Almaty 050040, Kazakhstan; 2 Department of Pre-University Education, Farabi University, Almaty 050040, Kazakhstan; 3 Department of Molecular Biology and Genetics, Faculty of Biology and Biotechnology, Al-Farabi Kazakh National University, Almaty 050040, Kazakhstan; 4 Department of Biotechnology, Mukhtar Auezov South Kazakhstan University, Shymkent 160001, Kazakhstan; 5 LLP “Institute of Coal Chemistry and Technology”, Astana 010000, Kazakhstan; 6 Institute of Crop Research, Xinjiang Academy of Agricultural Sciences, Urumqi 830091, China

**Keywords:** gamma irradiation, spring wheat, mutant lines, biological yield, iron, zinc, phytic acid, mineral bioavailability, principal component analysis, biofortification

## Abstract

Gamma irradiation is an effective mutagenesis approach for developing wheat lines with improved agronomic performance and enhanced nutritional quality. This study evaluated the effects of gamma irradiation on biological yield, protein content, iron (Fe), zinc (Zn), phytic acid concentration, and mineral bioavailability in mutant lines derived from three spring wheat cultivars (Zhenis, Almaken, and Eritrospermum-35). Mutant lines developed following 100 Gy and 200 Gy gamma irradiation were compared with their respective parental cultivars. Biological yield, protein, Fe and Zn concentrations, phytic acid content, and phytate-to-iron (Phy:Fe) and phytate-to-zinc (Phy:Zn) molar ratios were determined. Gamma irradiation affected biological yield and enhanced iron and zinc accumulation while reducing phytic acid concentration and phytate molar ratios, resulting in improved potential mineral bioavailability. In general, mutant lines developed at 100 Gy exhibited the most favorable combination of high biological yield, increased Fe and Zn concentrations, reduced phytic acid content, and improved mineral bioavailability. Principal component analysis (PCA) clearly separated mutant lines from their parental cultivars and identified superior genotypes associated with high biological yield, enhanced mineral accumulation, and lower phytate-related indices. Overall, these findings demonstrate that gamma irradiation, particularly at 100 Gy, is an effective strategy for improving biological yield, iron and zinc accumulation, and potential mineral bioavailability in spring wheat mutant lines, providing valuable genetic resources for future wheat biofortification programs.

## Simple Summary

Improving the nutritional quality of wheat while maintaining high grain yield is an important objective for sustainable agriculture and global food security. In this study, gamma irradiation was used to develop spring wheat mutant lines with enhanced agronomic and nutritional characteristics. The mutant lines were evaluated for grain yield, protein content, iron and zinc accumulation, phytic acid concentration, and mineral bioavailability. Several mutant lines showed increased grain yield together with higher iron and zinc concentrations and lower phytate-to-mineral molar ratios, indicating potentially improved mineral bioavailability. These findings suggest that gamma irradiation can effectively generate valuable genetic variation and that the selected mutant lines may serve as promising breeding materials for developing nutritionally improved wheat cultivars.

## 1. Introduction

Wheat (*Triticum aestivum* L.) is one of the world’s most widely cultivated cereal crops and serves as a staple food for more than one-third of the global population, providing approximately 20% of the daily dietary energy and protein intake [1,2,3]. Beyond its essential contribution to food security, wheat is an important dietary source of micronutrients, particularly iron (Fe) and zinc (Zn), which are indispensable for numerous biological processes, including oxygen transport, immune function, enzymatic activity, and normal human growth and development [4,5]. However, despite substantial advances in wheat breeding that have significantly increased grain productivity, improvements in grain nutritional quality have been comparatively limited [6]. Consequently, iron and zinc deficiencies remain among the most widespread forms of hidden hunger, affecting more than two billion people worldwide, particularly in regions where wheat constitutes a major component of the daily diet [7,8,9]. This global nutritional challenge has stimulated considerable interest in developing wheat cultivars with enhanced micronutrient concentration and improved mineral bioavailability through sustainable breeding strategies [9,10,11].

Biofortification has emerged as one of the most promising and cost-effective approaches for alleviating micronutrient malnutrition. Unlike conventional food fortification or dietary supplementation, biofortification increases the nutritional value of crops during plant growth through plant breeding, agronomic practices, mutation breeding, or modern biotechnological approaches [10,11,12,13]. In wheat, biofortification efforts primarily aim to increase grain Fe and Zn concentrations while maintaining high grain yield and desirable processing quality [12,13,14]. However, improving mineral concentration alone does not necessarily enhance nutritional value because the proportion of minerals available for intestinal absorption is strongly influenced by their bioavailability. Therefore, modern wheat improvement programs increasingly emphasize the simultaneous enhancement of mineral concentration and mineral bioavailability rather than focusing solely on total mineral accumulation [14].

Phytic acid (myo-inositol hexakisphosphate) is recognized as the principal antinutritional factor limiting mineral bioavailability in cereal grains [15]. It serves as the primary storage form of phosphorus in wheat seeds but also exhibits a strong affinity for divalent metal ions, including Fe^2+^ and Zn^2+^, forming insoluble phytate complexes that substantially reduce mineral absorption in the human gastrointestinal tract [16,17]. Consequently, reducing phytic acid concentration while increasing grain Fe and Zn content represents an effective strategy for improving the nutritional quality of wheat. For this reason, phytate-to-iron (Phy:Fe) and phytate-to-zinc (Phy:Zn) molar ratios are widely accepted indicators of mineral bioavailability, with lower values indicating greater potential mineral absorption and improved nutritional quality [18,19,20].

Gamma irradiation has become one of the most widely applied physical mutagens in plant mutation breeding because of its ability to generate novel genetic variation without introducing foreign DNA into the plant genome [21,22,23,24]. Ionizing radiation induces a wide range of genetic alterations, including nucleotide substitutions, deletions, chromosomal rearrangements, and modifications in gene expression, thereby creating new allelic combinations that may improve agronomic performance, grain quality, nutritional composition, and tolerance to environmental stresses [22,23,24,25]. Unlike conventional hybridization, mutation breeding enables the improvement of elite cultivars while preserving most of their favorable genetic background, making it an efficient approach for crop improvement [23,24,25,26].

Numerous studies have demonstrated that the effectiveness of gamma irradiation depends strongly on both genotype and irradiation dose [27,28,29,30]. Moderate doses generally stimulate beneficial genetic variation and may improve plant growth, grain yield, and nutritional quality, whereas excessive irradiation can cause severe DNA damage, physiological stress, and reduced plant performance [28,29,30,31]. Consequently, selecting an appropriate irradiation dose is essential for maximizing favorable mutations while minimizing undesirable genetic changes. In wheat breeding programs, irradiation doses ranging from 100 to 200 Gy have frequently been reported to produce valuable mutant populations with improved agronomic and nutritional characteristics [25,30,32]. Nevertheless, the magnitude of these improvements remains highly genotype-dependent, highlighting the importance of evaluating individual mutant lines under field conditions.

In recent years, wheat improvement programs have increasingly focused on combining high grain productivity with enhanced nutritional quality. Grain protein content, iron and zinc concentrations, phytic acid content, and mineral bioavailability are now considered key selection criteria for the development of nutritionally superior wheat cultivars [13,30,31,32,33]. However, these traits are quantitatively inherited and are influenced by complex interactions among genotype, environmental conditions, and physiological mechanisms regulating nutrient uptake, translocation, and storage within developing grains [32,33]. Moreover, increases in grain yield may dilute mineral concentrations, whereas reductions in phytic acid can substantially improve mineral bioavailability without markedly changing total mineral content. Therefore, simultaneous evaluation of agronomic performance and nutritional quality provides a more comprehensive assessment of breeding value than the evaluation of individual traits alone [33].

To better understand the relationships among these complex traits, multivariate statistical approaches have become increasingly important in crop improvement research. Principal component analysis (PCA) enables the identification of major sources of phenotypic variation and reveals relationships among agronomic and nutritional traits, facilitating the identification of superior genotypes [34,35,36]. The application of multivariate approaches improves selection efficiency and provides a more comprehensive understanding of the overall performance of mutant germplasm.

Despite the substantial progress achieved in wheat mutation breeding, relatively few studies have comprehensively investigated the combined effects of gamma irradiation on grain yield, protein content, iron and zinc accumulation, phytic acid concentration, and mineral bioavailability within the same mutant population [37]. Most previous studies have focused on individual agronomic or nutritional traits, whereas integrated assessments encompassing productivity, micronutrient accumulation, antinutritional factors, and mineral bioavailability remain limited. Furthermore, information on gamma irradiation-induced mutant lines developed from Kazakh spring wheat cultivars is still scarce, despite their potential value as genetic resources for wheat biofortification and breeding programs [38,39,40,41].

To our knowledge, no previous study has comprehensively evaluated gamma irradiation-derived spring wheat mutant lines from Kazakhstan by simultaneously integrating grain yield, grain protein content, iron and zinc accumulation, phytic acid concentration, phytate-to-mineral molar ratios, and multivariate statistical analysis, including principal component analysis (PCA). Such an approach provides a more comprehensive understanding of the relationships between agronomic performance and nutritional quality and facilitates the identification of superior mutant genotypes with enhanced breeding potential.

Therefore, the objective of this study was to evaluate the effects of 100 Gy and 200 Gy gamma irradiation on biological yield, protein content, iron and zinc accumulation, phytic acid concentration, and mineral bioavailability in spring wheat mutant lines derived from three Kazakh cultivars. In addition, PCA was employed to investigate the relationships among agronomic and nutritional traits and to identify promising mutant lines for wheat biofortification. The findings of this study provide new evidence supporting the application of gamma irradiation as an effective mutation breeding strategy for developing nutritionally improved wheat germplasm with enhanced mineral bioavailability while maintaining desirable agronomic performance.

## 2. Materials and Methods

### 2.1. Plant Material and Gamma Irradiation

Three spring wheat cultivars, namely Zhenis, Almaken, and Erythrospermum-35 (*Triticum aestivum* L.), and their corresponding gamma-irradiation-derived mutant lines were evaluated in this study. The seeds of the parental cultivars were obtained from the Kazakh Research Institute of Agriculture and Crop Production, Almaty, Kazakhstan. Dry seeds of the parental cultivars were exposed to gamma irradiation at doses of 100 Gy and 200 Gy using a ^60^Co gamma source at the Kazakh Nuclear Center. These doses were selected based on previous studies indicating that 100–200 Gy can be effective for inducing desirable variation in bread wheat [42]. The irradiation treatment was performed under controlled conditions, with the seeds maintained in a dry state during exposure. For each wheat cultivar, 2000 dry seeds were treated at each irradiation dose (100 and 200 Gy) [38]. Following field establishment, emergence rates varied among cultivars and irradiation doses. For Zhenis, emergence ranged from 80–90% at 100 Gy and 80–100% at 200 Gy. For Almaken and Erythrospermum-35, emergence ranged from 80–90% at 100 Gy and 85–100% at 200 Gy. Accordingly, the proportion of seeds that failed to emerge ranged from 10–20% at 100 Gy and from 0–20% at 200 Gy, depending on the cultivar. The irradiated seeds were not germinated prior to planting and were subsequently sown directly under field conditions one month after irradiation. The exposure time was determined according to the calibrated dose rate of the ^60^Co gamma source to achieve the required absorbed doses of 100 and 200 Gy. The distance between the radiation source and the seed material was maintained according to the standard operating conditions of the irradiation facility. Non-irradiated seeds of the corresponding parental cultivars were maintained as controls and were subsequently handled under the same field conditions as the irradiated material.

**Figure 1:**
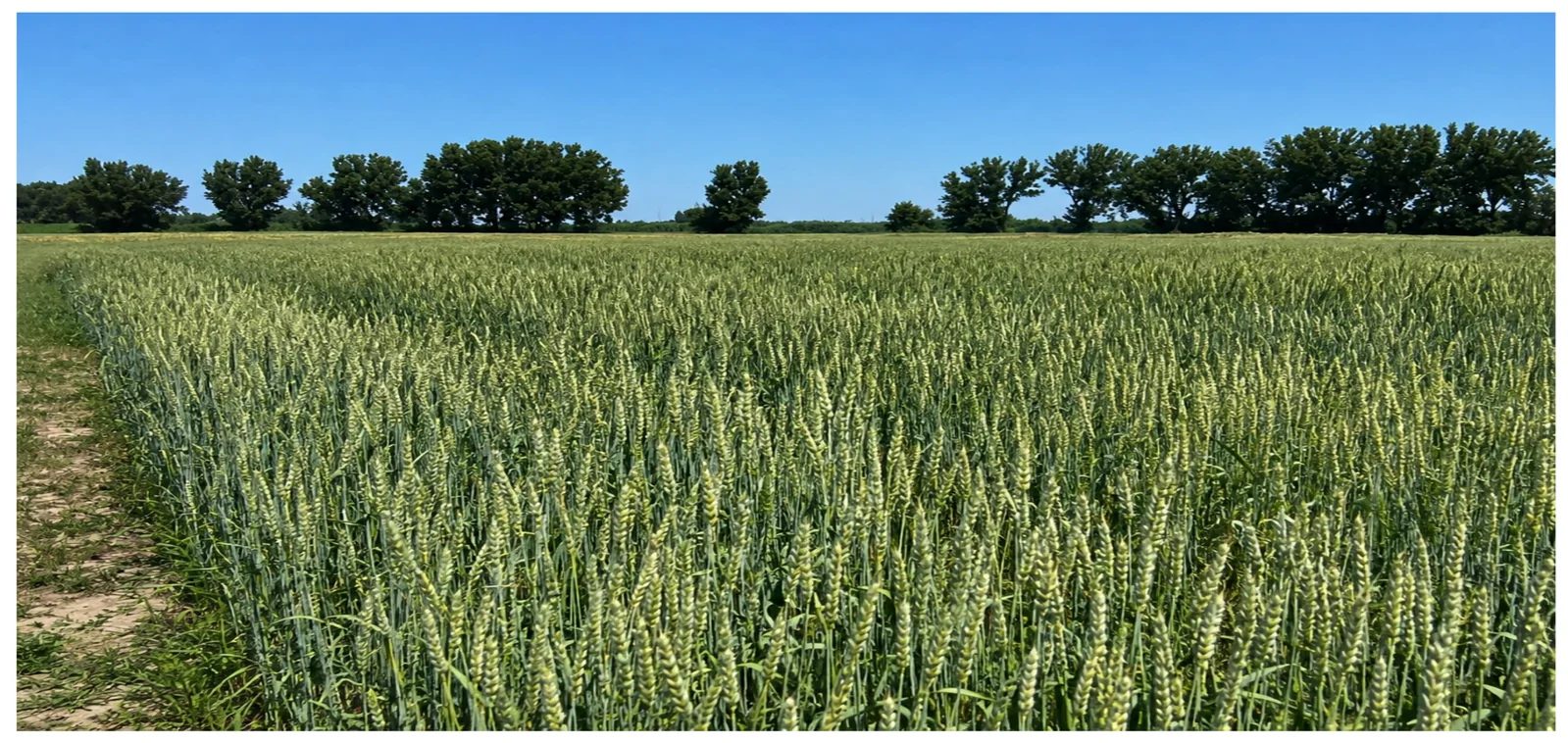
Field-grown wheat plants of the parental cultivars and gamma-irradiation-induced mutant lines.

The irradiated seeds were subsequently advanced through successive generations under field conditions, and promising mutant lines were selected based on agronomic performance and yield-related traits. Fifteen promising mutant lines were selected for each cultivar × irradiation-dose combination for further evaluation. The M_1_ generation was established from the irradiated seeds, and individual plants were evaluated and selected for further propagation. In subsequent generations, individual-plant and progeny-based selection was performed to identify lines exhibiting stable and improved agronomic characteristics. The selection process was continued through successive generations, and promising mutant lines were advanced to the M_5_ generation for further evaluation. During the M_1_–M_3_ generations, selection was based exclusively on phenotypic and agronomic characteristics, including plant development and yield-related traits. Growth- and yield-related traits evaluated during field selection included the number of stems per plant, main stem length, number of spikes per plant, main spike length, number and weight of grains per main spike, and total grain number and grain weight per plant. These traits were primarily used for phenotypic evaluation and selection of promising mutant lines and were not included among the main traits reported in the present study. In the M_3_ and M_4_ generations, promising mutant plants and lines were further selected based on grain weight per spike (GWS) and grain weight per plant (GWP), using the corresponding parental cultivar as a reference [40]. No biochemical or molecular analyses were performed during these early generations. Beginning with the M_4_ generation, biochemical analyses were introduced to further characterize the selected mutant lines. From the M_5_ generation onward, biochemical and molecular analyses were progressively incorporated to provide additional characterization of the advanced mutant lines. This stage was selected because phenotypic and genetic characteristics are expected to become more stable through successive generations, allowing more reliable characterization of genetically distinct mutant lines. Molecular analyses were therefore performed from the M_5_ generation to investigate genetic diversity and molecular characteristics among the selected mutants. Not all mutant lines were subjected to the same biochemical and molecular analyses; the analyses performed depended on the genetic diversity and specific characteristics of individual lines. Thus, different mutant lines were selected for different downstream biochemical and molecular analyses. The selected mutant lines were compared with their respective parental cultivars under the same field conditions.Field experiments were conducted at the experimental field of the Kazakh Research Institute of Agriculture and Crop Production, located near Almaty, Kazakhstan (43°15′ N, 76°54′ E, 550 m above sea level), under typical continental climatic conditions of southeastern Kazakhstan. The irradiated seeds were sown one month after irradiation. The parental cultivars and their corresponding mutant lines were grown simultaneously under comparable environmental and agronomic conditions. The experimental site was characterized by brown medium-loam soil with a pH of approximately 7.5. The topsoil layer (0–20 cm) contained 2–2.6% humus, with total nitrogen ranging from 0.189 to 0.20%, mobile phosphorus from 16.3 to 17.8 mg kg^−1^, and exchangeable potassium from 451 to 498 mg kg^−1^. The field experiments were conducted under rainfed conditions, and no supplementary irrigation was applied during the growing season. No mineral fertilizers were applied during the growing season. The field trials were established using a randomized complete block design with three biological replicates. Each experimental plot consisted of three rows, 20 m in length and 1.20 m in width, with 20 cm between rows and 25 cm between seeds within each row. The experiments were managed according to locally recommended agronomic practices for spring wheat production. The field-grown wheat plants of the parental cultivars and gamma-irradiation-induced mutant lines are shown in Figure 1.The experimental design included two factors: wheat genotype and gamma irradiation dose. The genotype factor consisted of three levels (Zhenis, Almaken, and Erythrospermum-35), while the irradiation factor consisted of three levels (0, 100, and 200 Gy). Thus, the experiment comprised nine genotype × irradiation-dose combinations, with three biological replicates for each combination. The treatment combinations were randomly assigned within each block of the randomized complete block design.

### 2.2. Determination of Thousand-Grain Weight (TGW)

Thousand-grain weight (TGW) was determined for each genotype by weighing three sets of 100 randomly selected mature grains. The mean weight of the three 100-grain samples was multiplied by 10 to calculate the thousand-grain weight. Grain weight was measured using a standard laboratory balance, and the results were expressed in grams (g). Three biological replicates were analyzed for each genotype, and the results were expressed as mean ± standard deviation (SD) [40].

### 2.3. Determination of Grain Protein Content

Grain protein concentration was determined using a ZX50 Portable Grain Analyzer (ZX Instruments, Hagerstown, MD, USA) based on near-infrared reflectance spectroscopy (NIRS). Prior to analysis, grain samples were cleaned and equilibrated to room temperature. Three biological replicates were analyzed, with 25 grains used per sample. Grain protein content was measured according to the manufacturer’s calibration protocol, and the results were expressed as percentage (%) on a dry weight basis [40]. The experimental design consisted of three treatments (control, 100 Gy, and 200 Gy), three genotypes, and three biological replicates (3 treatments × 3 genotypes × 3 replicates).

### 2.4. Determination of Iron and Zinc Concentrations

Grain samples collected from the selected mutant lines and their corresponding parental cultivars were thoroughly cleaned with deionized water, oven-dried to constant weight at 65–70 °C, and finely ground using a laboratory mill (Retsch MM400, Hamburg, Germany). Approximately 0.2 g of each powdered sample was transferred into digestion vessels for mineral analysis. Samples were digested using a mixture of concentrated nitric acid (HNO_3_, 65%) and hydrogen peroxide (H_2_O_2_, 30%) in a 5:1 (*v*/*v*) ratio. The digestion procedure was carried out in a programmable digestion system following a gradual temperature program until complete mineralization was achieved. After cooling, the digested solutions were diluted to a final volume of 20 mL with ultrapure water. Iron (Fe) and zinc (Zn) concentrations were quantified using flame atomic absorption spectrometry (AAS) (Analytik Jena NovaAA 350, Jena, Germany). Certified reference materials and reagent blanks were included throughout the analytical procedure to verify measurement accuracy. Three independent biological replicates were analyzed for each genotype, and the mean values were used for subsequent analyses [41].

### 2.5. Determination of Phytic Acid Content

Phytic acid concentration (PA) was determined using the Megazyme Phytic Acid Assay Kit (Megazyme International, Bray, Ireland) according to the manufacturer’s protocol with slight modifications. Briefly, 2.0 g of finely ground grain flour was transferred into centrifuge tubes and extracted with 0.66 M hydrochloric acid (HCl) under continuous shaking at room temperature for 8 h. The extracts were centrifuged at 13,000 rpm for 8 min, and the resulting supernatants were collected for analysis. Aliquots of the extracts were subjected to enzymatic hydrolysis using the reagents supplied in the assay kit. The absorbance of each reaction mixture was measured at 655 nm using an Eppendorf BioPhotometer Plus (Hamburg, Germany). Phytic acid concentration was calculated according to the manufacturer’s equations and expressed as mg g^−1^ dry weight. All samples were analyzed in triplicate [41].

### 2.6. Calculation of Phy:Fe and Phy:Zn Molar Ratios

To estimate mineral bioavailability, the molar ratios between phytic acid and the two essential micronutrients were calculated. The concentrations of phytic acid, iron, and zinc were first converted into molar quantities using their respective molecular or atomic weights (660.04 g mol^−1^ for phytic acid, 55.85 g mol^−1^ for iron, and 65.38 g mol^−1^ for zinc) [41]. The Phy:Fe and Phy:Zn molar ratios were then obtained by dividing the molar concentration of phytic acid by the corresponding molar concentration of each mineral. Lower Phy:Fe and Phy:Zn ratios were interpreted as indicators of improved mineral bioavailability.

### 2.7. Statistical Analysis

All experimental data were analyzed using GenStat (10th Edition, VSN International, Hemel Hempstead, UK) and R software (version 4.6.0). Prior to statistical analysis, data normality and homogeneity of variances were assessed using the Shapiro–Wilk and Levene’s tests, respectively. The effects of wheat genotype, gamma irradiation dose, and their interaction were evaluated using a two-way analysis of variance (ANOVA) based on the factorial experimental design. The statistical model included genotype, irradiation dose, genotype × irradiation dose interaction, and block effects, with biological replicates treated as blocks. When significant effects were detected, mean comparisons were performed using Dunnett’s multiple comparison test, with irradiated mutant treatments compared directly with their corresponding non-irradiated parental controls. Pearson correlation analysis and principal component analysis (PCA) were performed to investigate relationships among agronomic, nutritional, mineral, and mineral bioavailability traits. For PCA, mean values obtained from three biological replicates were used. F-values, degrees of freedom (df), and corresponding *p*-values were reported for the main effects of genotype and irradiation dose, as well as their interaction. All measurements were conducted using three biological replicates and are presented as mean ± standard deviation (SD). Differences were considered statistically significant at *p* < 0.05.

## 3. Results

### 3.1. Effect of Gamma Irradiation on Biological Yield and Thousand-Grain Weight

The effects of gamma irradiation on biological yield and thousand-grain weight (TGW) are presented in Table 1. Both traits varied among the three wheat genotypes and irradiation treatments. In the Zhenis genotype, biological yield was 17.25 ± 0.91 t ha^−1^ in the control treatment and increased to 28.65 ± 3.81 t ha^−1^ following 100 Gy irradiation, corresponding to a 66.1% increase relative to the control. At 200 Gy, biological yield was 19.51 ± 2.91 t ha^−1^, representing a 13.1% increase.

TGW increased from 38.08 ± 3.67 g in the control to 41.54 ± 4.81 g and 44.25 ± 9.24 g following 100 and 200 Gy irradiation, respectively. In the Almaken genotype, biological yield was 21.10 ± 2.40 t ha^−1^ in the control treatment and increased to 35.73 ± 1.34 t ha^−1^ after 100 Gy irradiation, corresponding to a 69.3% increase relative to the control. At 200 Gy, biological yield was 23.96 ± 1.33 t ha^−1^, representing a 13.2% increase. TGW increased from 34.10 ± 1.17 g in the control to 48.81 ± 4.87 g and 47.27 ± 6.65 g following 100 and 200 Gy irradiation, respectively.

In Eritospermum-35, biological yield increased from 11.33 ± 2.20 t ha^−1^ in the control to 26.92 ± 3.87 t ha^−1^ and 27.14 ± 3.95 t ha^−1^ following 100 and 200 Gy irradiation, respectively, corresponding to increases of 137.6% and 139.5% relative to the control. TGW increased from 31.10 ± 1.25 g in the control to 46.98 ± 8.27 g and 48.75 ± 5.74 g following 100 and 200 Gy irradiation, respectively.

Overall, biological yield and TGW were higher in the irradiated treatments than in the respective controls, although the magnitude of the response varied among genotypes and irradiation doses. The response to gamma irradiation was genotype-dependent, with 100 Gy producing a greater increase in biological yield in Zhenis and Almaken, whereas Erythrospermum-35 maintained a higher yield at 200 Gy.

### 3.2. Grain Protein and Mineral Accumulation

**Figure 2:**
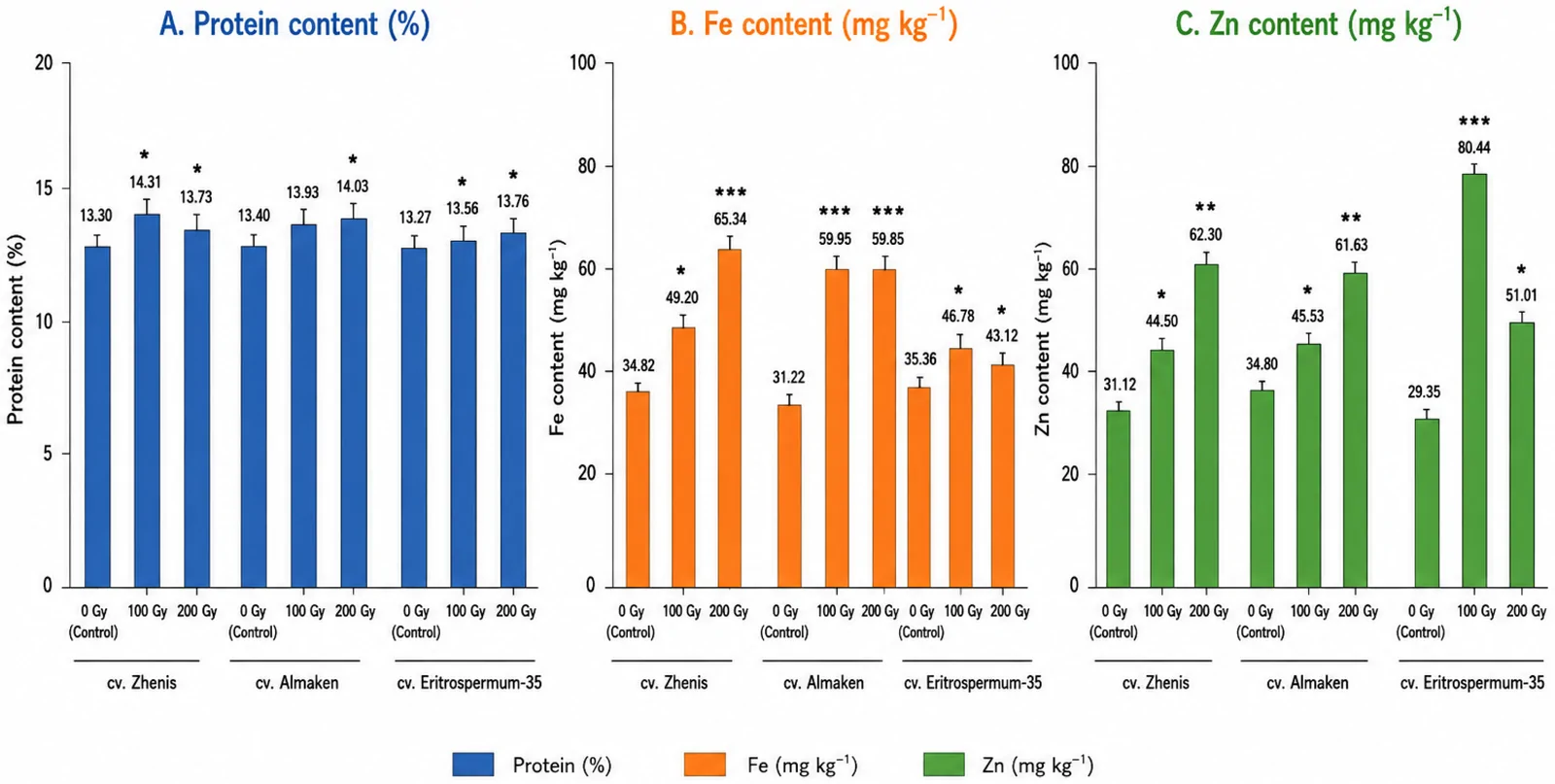
Grain protein (**A**), iron (Fe) (**B**), and zinc (Zn) (**C**) contents of control and gamma-irradiated wheat mutant lines. Data are presented as mean ± SD (n = 3 biological replicates). Asterisks indicate significant differences compared with the corresponding non-irradiated parental control according to Dunnett’s multiple comparison test (* *p* < 0.05, ** *p* < 0.01, *** *p* < 0.001). 0 Gy (Control)—non-irradiated parental line.

The effects of gamma irradiation on grain protein, iron (Fe), and zinc (Zn) concentrations in the selected wheat genotypes are presented in Figure 2. Overall, gamma irradiation improved grain nutritional quality by increasing protein and mineral concentrations, although the magnitude of the response differed among genotypes and irradiation doses.

Protein content showed relatively small but consistent increases in all mutant lines compared with their respective controls. In the Zhenis genotype, protein content increased from 13.30% in the control to 14.31% and 13.73% following 100 Gy and 200 Gy irradiation, respectively. Similar trends were observed in Almaken and Eritrospermum-35, indicating that gamma irradiation had a modest but positive effect on grain protein accumulation.

In contrast, the response of mineral accumulation was more pronounced than that of protein. Iron concentration increased substantially in all mutant lines relative to the controls. The highest Fe concentration (65.34 mg kg^−1^) was recorded in the 200 Gy-treated Zhenis mutant line, while both irradiated Almaken mutant lines also exhibited markedly higher Fe concentrations than the corresponding control. In Eritrospermum-35, Fe concentration increased from 35.36 mg kg^−1^ in the control to 46.78 mg kg^−1^ following the 100 Gy treatment.

Zinc accumulation showed a genotype-dependent response to gamma irradiation. The greatest Zn concentration (80.44 mg kg^−1^) was observed in the 100 Gy-treated Eritrospermum-35 mutant line, representing the highest level among all evaluated genotypes. In addition, both irradiated Zhenis and Almaken mutant lines exhibited considerably higher Zn concentrations than their respective controls, although the response differed between irradiation doses.

Collectively, these results demonstrate that gamma irradiation enhanced grain nutritional quality by increasing protein, iron, and zinc concentrations, with mineral accumulation showing a substantially stronger response than protein content. The greatest improvements were generally observed at 100 Gy, although genotype-dependent variation was evident across all evaluated traits.

### 3.3. Phytic Acid Content and Phytate-to-Mineral Ratios

**Figure 3:**
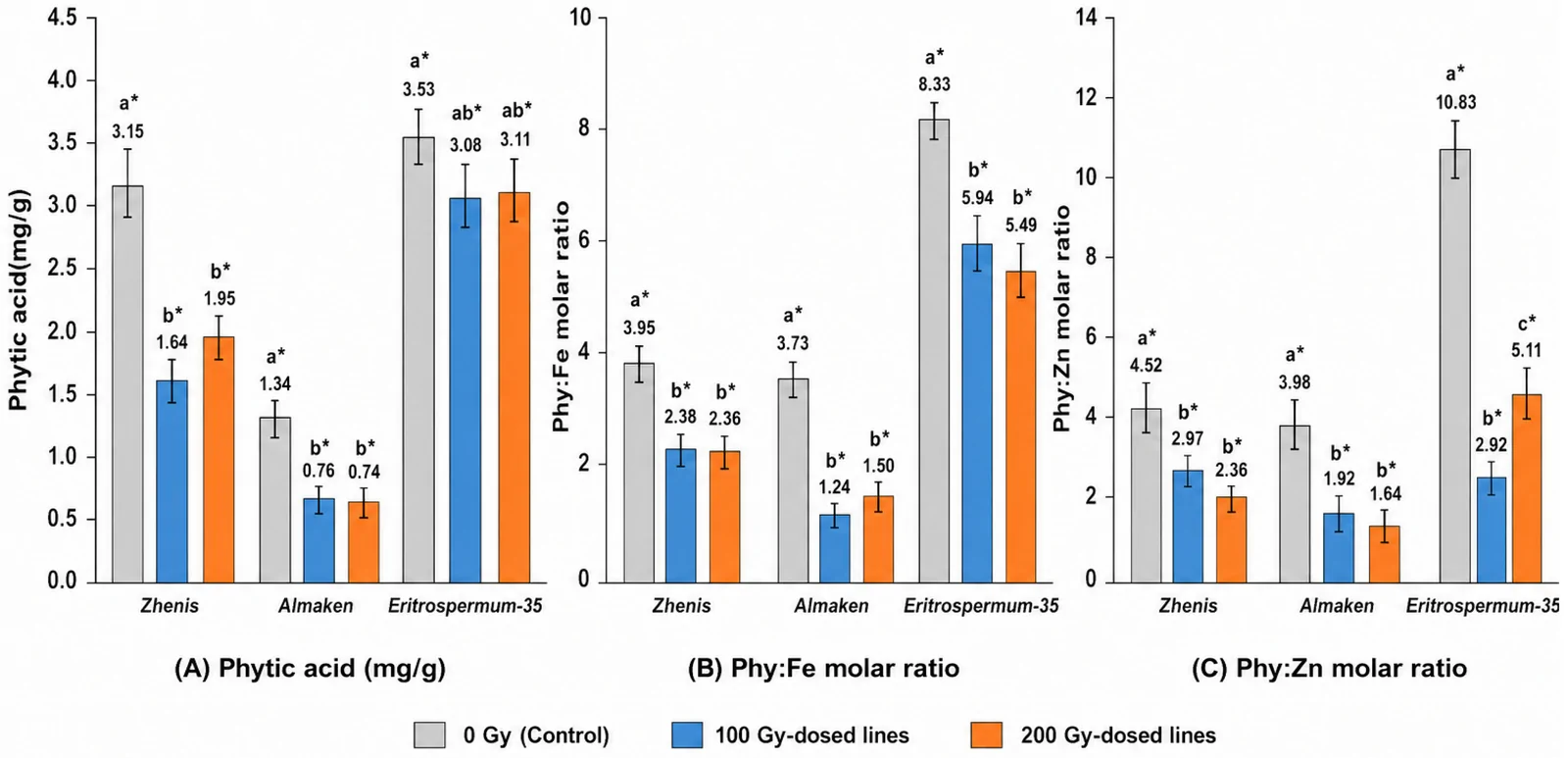
Effect of gamma irradiation dose on (**A**) phytic acid concentration (mg/g), (**B**) Phy:Fe molar ratio, and (**C**) Phy:Zn molar ratio in spring wheat cultivars and gamma irradiation-induced mutant lines. Bars represent mean ± SD (n = 3 biological replicates). Different lowercase letters indicate significant differences among irradiation doses within the same genotype according to one-way ANOVA followed by Tukey’s HSD test (*p* < 0.05). Asterisks (*) indicate significant differences compared with the corresponding 0 Gy control.

Gamma irradiation affected phytic acid concentration and the phytate-to-iron (Phy:Fe) and phytate-to-zinc (Phy:Zn) molar ratios in all three spring wheat genotypes, although the magnitude of the response varied depending on genotype and irradiation dose. In general, the irradiated mutant lines showed lower phytic acid concentrations and lower Phy:Fe and Phy:Zn molar ratios than their corresponding parental cultivars, suggesting a more favorable phytate-related mineral nutritional profile (Figure 3).

Among the evaluated genotypes, the greatest reduction in phytic acid concentration was observed in the Almaken mutant lines. The phytic acid concentration decreased from 1.34 mg g^−1^ in the parental cultivar to 0.76 and 0.74 mg g^−1^ following 100 and 200 Gy irradiation, respectively. In Zhenis, the corresponding values decreased from 3.15 mg g^−1^ in the control to 1.64 and 1.95 mg g^−1^ after 100 and 200 Gy irradiation, respectively. Eritospermum-35 showed a similarly pronounced response, with phytic acid concentration decreasing from 3.53 mg g^−1^ in the control to 3.08 and 3.11 mg g^−1^ following 100 and 200 Gy treatments, respectively. The different lowercase letters shown in Figure 3 indicate significant differences among irradiation treatments within the same genotype.

A similar pattern was observed for the Phy:Fe molar ratio. In Zhenis, the ratio decreased from 3.95 in the control to 2.38 and 2.36 following 100 and 200 Gy irradiation, respectively. In Almaken, the ratio decreased from 3.73 in the control to 1.24 and 1.50 following 100 and 200 Gy irradiation, respectively. Eritospermum-35 exhibited the highest Phy:Fe molar ratio in the control (8.33), which decreased to 5.94 and 5.49 following 100 and 200 Gy irradiation, respectively. Thus, both irradiation treatments resulted in lower Phy:Fe ratios than those observed in the corresponding parental cultivars.

The Phy:Zn molar ratio also decreased following gamma irradiation compared with the parental controls. In Zhenis, the ratio declined from 4.52 in the control to 2.97 and 2.36 after 100 and 200 Gy irradiation, respectively. In Almaken, the ratio decreased from 3.98 to 1.92 and 1.64 following 100 and 200 Gy treatments, respectively. In Eritospermum-35, the Phy:Zn molar ratio decreased from 10.83 in the control to 2.92 following 100 Gy irradiation and to 5.11 following 200 Gy irradiation.

The observed reductions in phytic acid concentration and Phy:Fe and Phy:Zn molar ratios indicate substantial changes in the phytate-related mineral nutritional profile of the gamma-irradiated mutant lines. Lower Phy:Fe and Phy:Zn molar ratios are generally associated with a lower potential for phytate-mediated inhibition of iron and zinc absorption. Therefore, the reductions observed in the present study may suggest a more favorable nutritional profile of the mutant lines. However, because direct iron and zinc absorption or bioavailability was not measured in this study, these findings should be interpreted as indirect indicators rather than direct evidence of improved mineral bioavailability.

Overall, the results demonstrate that gamma irradiation modified phytic acid accumulation and phytate-to-mineral relationships in spring wheat, with the strongest reductions in phytic acid and Phy:Fe and Phy:Zn molar ratios generally observed in the Almaken mutant lines. These changes may contribute to the nutritional potential of the selected mutant lines; however, direct in vitro or in vivo mineral absorption assays would be required to confirm their effects on iron and zinc bioavailability.

### 3.4. Principal Component Analysis (PCA)

**Figure 4:**
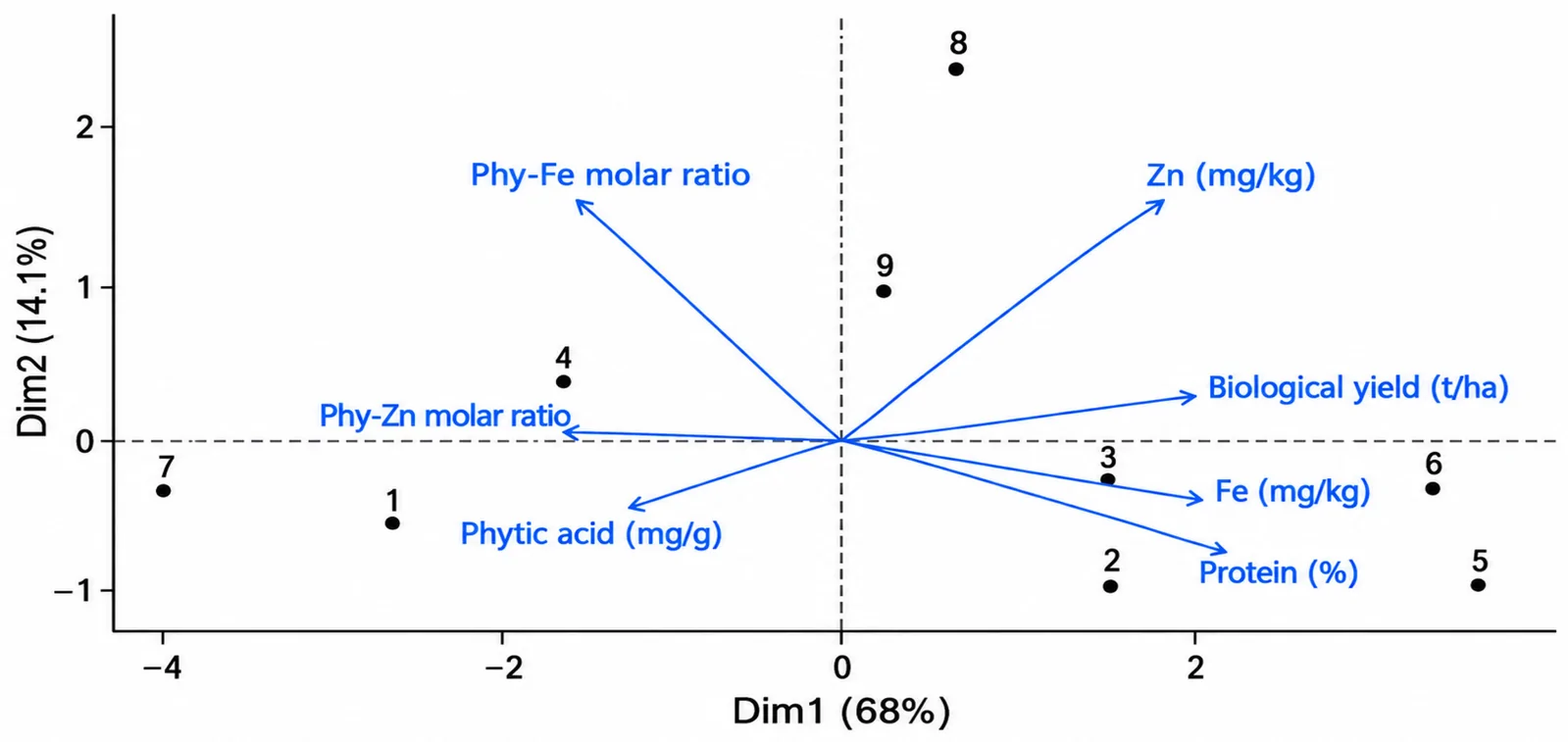
PCA biplot illustrating the relationships among agronomic and nutritional traits of spring wheat cultivars and gamma-induced mutant lines: (1) cv. Zhenis, (2) Zhenis 100 Gy-dosed lines, (3) Zhenis 200 Gy-dosed lines, (4) cv. Almaken, (5) Almaken 100 Gy-dosed lines, (6) Almaken 200 Gy-dosed lines, (7) cv. Eritrospermum-35, (8) Eritrospermum-35 100 Gy-dosed lines, and (9) Eritrospermum-35 200 Gy-dosed lines.

Principal component analysis (PCA) was performed to investigate the relationships among agronomic and nutritional traits of the evaluated spring wheat genotypes (Figure 4). The first two principal components explained 82.1% of the total variation, with PC1 accounting for 68.0% and PC2 for 14.1%, indicating that the biplot adequately represented the variability among the studied genotypes. PC1 primarily separated the genotypes according to biological yield, grain protein content, Fe and Zn concentrations, whereas phytic acid concentration together with the Phy:Fe and Phy:Zn molar ratios were associated with the opposite direction of the axis.

Biological yield, protein content, Fe concentration, and Zn concentration were positively associated, as indicated by the similar orientation of their vectors along the positive side of PC1. In contrast, phytic acid concentration and the Phy:Fe and Phy:Zn molar ratios were positioned in the opposite direction, indicating negative associations with biological yield and mineral accumulation.

The 100 Gy-treated Eritrospermum-35 mutant line showed the highest positive score on PC2 and was closely associated with increased Zn concentration, whereas the irradiated Zhenis and Almaken mutant lines clustered with higher biological yield, protein content, and Fe concentration.

The distribution of individual genotypes further demonstrated genotype-dependent responses to gamma irradiation. The 100 Gy-treated Eritrospermum-35 mutant line showed the highest positive score on PC2 and was closely associated with increased Zn concentration, whereas the irradiated Zhenis and Almaken mutant lines clustered with higher biological yield, protein content, and Fe concentration.

Overall, the PCA biplot demonstrated that improved agronomic performance and mineral accumulation were associated with reduced phytic acid concentration and lower phytate-to-mineral molar ratios, clearly distinguishing the irradiated mutant lines from their parental cultivars.

Two-way ANOVA revealed significant effects of wheat genotype, gamma irradiation dose, and their interaction on most of the measured traits (Table 2). Biological yield was significantly affected by genotype (F(2,16) = 54.11, *p* = 7.57 × 10^−8^), irradiation dose (F(2,16) = 304.62, *p* = 1.84 × 10^−13^), and their interaction (F(4,16) = 37.15, *p* = 6.56 × 10^−8^). Similarly, Fe concentration was significantly affected by genotype (F(2,16) = 62.71, *p* = 2.68 × 10^−8^), irradiation dose (F(2,16) = 419.65, *p* = 1.50 × 10^−14^), and the genotype × dose interaction (F(4,16) = 58.64, *p* = 2.35 × 10^−9^). Significant effects of genotype, irradiation dose, and their interaction were also observed for Zn concentration, phytic acid concentration, Phy:Fe molar ratio, and Phy:Zn molar ratio (Table 2). Protein content was not significantly affected by genotype (F(2,16) = 1.21, *p* = 0.323), irradiation dose (F(2,16) = 3.004, *p* = 0.078), or their interaction (F(4,16) = 0.36, *p* = 0.831). Block effects were generally not significant, except for Fe concentration (F(2,16) = 4.36, *p* = 0.031) and the Phy:Fe molar ratio (F(2,16) = 5.66, *p* = 0.014).

## 4. Discussion

Gamma irradiation has been widely recognized as an efficient mutation-breeding tool capable of generating novel genetic variation while preserving the desirable genetic background of elite cultivars [43,44,45,46]. In the present study, gamma irradiation significantly influenced biological yield, iron and zinc accumulation, phytic acid concentration, and mineral bioavailability, whereas grain protein content was only slightly affected. These findings indicate that irradiation primarily altered traits associated with mineral metabolism and nutrient partitioning rather than overall protein synthesis [46,47,48].

The superiority of the 100 Gy treatment observed in the present study agrees with the concept of radiation hormesis, whereby moderate doses may stimulate beneficial physiological and genetic responses, whereas higher doses may induce excessive oxidative stress, DNA damage, and metabolic disturbances [49,50,51,52]. In the present study, Zhenis and Almaken exhibited substantially greater increases in biological yield at 100 Gy (66.1% and 69.3%, respectively) than at 200 Gy (13.1% and 13.2%, respectively). In contrast, Eritospermum-35 maintained a pronounced increase in biological yield at both 100 and 200 Gy (137.6% and 139.5%, respectively), suggesting greater tolerance to the higher irradiation dose. The concurrent increase in TGW across irradiated treatments may have contributed to the observed improvement in biological yield. These genotype- and dose-dependent responses are consistent with previous findings showing that gamma irradiation at 100–200 Gy can induce substantial variation in grain yield and yield-related traits in bread wheat [39,40,42]. Such findings emphasize that the effectiveness of mutation breeding depends strongly on the interaction between irradiation dose and genetic background [46,47,48,53].

A major outcome of this study was the considerable increase in grain Fe and Zn concentrations following gamma irradiation. These improvements may be associated with irradiation-induced genetic changes that potentially affect processes involved in mineral uptake, long-distance transport, remobilization, and grain loading during seed development [54,55,56,57,58]. Similar effects of gamma irradiation on wheat phenotypic and agronomic traits have been reported by Ahumada-Flores et al. [59], who observed morphological and agronomic variation among M_2_ mutant plants following irradiation with 100–300 Gy. These findings are consistent with independent studies demonstrating that gamma irradiation can generate wheat mutant lines with improved agronomic and grain-quality traits, particularly at moderate irradiation doses [42]. In the present study, the increase in grain Fe and Zn concentrations suggests that irradiation-induced genetic variation may also contribute to enhanced micronutrient accumulation. This observation is consistent with previous research demonstrating substantial genetic variation for grain Fe and Zn concentrations in wheat and the feasibility of selecting lines with enhanced micronutrient accumulation [60]. These findings are in agreement with previous work on wheat mutant lines, where enhanced grain Fe and Zn accumulation was associated with differential expression of genes involved in iron homeostasis, suggesting that induced mutations may influence mineral uptake, transport, and remobilization processes [39]. Enhanced micronutrient accumulation is particularly important because Fe and Zn deficiencies remain among the most prevalent forms of hidden hunger worldwide [57,58]. Therefore, mutant lines combining improved grain yield with elevated micronutrient concentrations represent valuable genetic resources for wheat biofortification programs [56,57,58].

The beneficial effects of gamma irradiation observed in the present study may be associated with several physiological and molecular responses induced by radiation. Gamma irradiation can cause water radiolysis and the subsequent formation of reactive oxygen species (ROS), which may function as signaling molecules and activate antioxidant and stress-response pathways. At appropriate doses, these responses may contribute to enhanced stress tolerance and stimulation of plant growth and development. Gamma irradiation may also induce DNA damage and activate DNA repair and cellular regulatory mechanisms, potentially contributing to genetic variation and phenotypic changes in subsequent generations. Previous studies have reported dose-dependent changes in antioxidant activity, oxidative stress responses, and DNA damage and repair processes following gamma irradiation in plants. However, these molecular and physiological mechanisms were not directly investigated in the present study and are therefore discussed as possible explanations for the observed phenotypic responses [61,62,63].

Besides increasing mineral concentrations, gamma irradiation substantially reduced phytic acid concentration and consequently lowered the Phy:Fe and Phy:Zn molar ratios. Because phytic acid can inhibit iron and zinc absorption in cereal-based diets, lower phytate-to-mineral molar ratios are generally considered indicators of a potentially improved mineral bioavailability profile [64,65]. Therefore, the simultaneous increase in mineral concentrations and decrease in phytate observed in the present study may indicate a more favorable nutritional profile of the irradiated grains. A similar pattern has been reported in spring wheat mutant lines, where induced mutagenesis improved grain protein and micronutrient (Fe and Zn) contents while also affecting phytate-related nutritional traits [40]. However, mineral bioavailability was not directly assessed in this study, and these conclusions are based on established relationships reported in the literature rather than direct absorption measurements. Among the evaluated genotypes, Almaken showed the lowest phytic acid concentration and the lowest Phy:Fe and Phy:Zn molar ratios, whereas Erythrospermum-35 exhibited the highest zinc accumulation. These genotype-specific differences may be relevant for different breeding objectives.

The multivariate analyses provided additional evidence supporting the observed relationships among the measured traits. PCA demonstrated that grain yield, Fe concentration, Zn concentration, and protein content were positively associated, whereas phytic acid and phytate-to-mineral molar ratios were negatively correlated with these desirable traits. The multivariate analysis further supported the separation between parental cultivars and mutant lines, indicating that gamma irradiation generated distinct phenotypic profiles characterized by improved nutritional quality [66]. The agreement between univariate statistical analyses and PCA increases confidence in the robustness of the observed biological responses.

Overall, the present findings indicate that gamma irradiation, particularly at 100 Gy, represents a promising strategy for simultaneously improving grain yield, micronutrient accumulation, and traits associated with potential mineral bioavailability in spring wheat. The identified mutant lines provide valuable germplasm for wheat biofortification and may contribute to the development of nutritionally superior cultivars capable of helping alleviate iron and zinc deficiencies in human populations [59,60,61,62,63,64,65,66].

### Limitations and Future Perspectives

Although this study demonstrates the potential of gamma irradiation for improving wheat nutritional quality, several limitations should be acknowledged. The experiments were conducted using a limited number of mutant lines under a single environmental condition, and mineral bioavailability was estimated indirectly using phytate-to-mineral molar ratios rather than through in vitro or in vivo absorption assays [63,64]. Furthermore, the molecular mechanisms underlying the observed improvements remain to be elucidated. Future studies involving multi-location field trials, transcriptomic or genomic analyses, and direct assessments of mineral bioavailability would provide a more comprehensive understanding of the genetic basis, stability, and nutritional significance of these traits [65].

This study has several limitations that should be considered when interpreting the results. The experimental plot size was relatively small, and only a limited number of promising mutant lines were selected from the total treated seed population for further evaluation. Therefore, the observed genotype- and dose-dependent responses should be validated using larger populations, multi-location trials, and multiple growing seasons to confirm the stability of the identified traits.

## 5. Conclusions

This study demonstrated that gamma irradiation influenced agronomic performance and nutritional quality-related traits in spring wheat, with responses varying among genotypes and irradiation doses. Under the conditions of the present study, the 100 Gy treatment generally produced the most pronounced improvements in biological yield, Fe and Zn accumulation, and traits associated with potential mineral bioavailability. Gamma irradiation also reduced phytic acid concentration and the Phy:Fe and Phy:Zn molar ratios, which are commonly used as indirect indicators of potential mineral bioavailability. Among the evaluated genotypes, Almaken exhibited the most favorable phytate-to-mineral ratio profile, whereas Eritrospermum-35 showed the greatest increase in grain Zn concentration. Multivariate analyses further supported the distinct responses of the mutant lines and the relationships among agronomic and nutritional traits. Overall, the identified mutant lines represent promising genetic resources for further wheat biofortification and breeding studies. However, direct in vitro or in vivo mineral absorption assays and multi-environment field evaluations are required to validate the actual bioavailability and stability of these traits before practical application in cultivar development.

## Figures and Tables

**Table 1:** Effect of gamma irradiation on the biological yield of selected wheat mutant lines.

Genotype	Dose (Gy)	TGW (g)	Biological Yield (t/ha)	Increase vs. Control (%)
Zhenis control	0	38.08 ± 3.67	17.25 ± 0.91	0
Zhenis mutant lines	100	41.54 ± 4.81	28.65 ± 3.81	66.1 **
Zhenis mutant lines	200	44.25 ± 9.24	19.51 ± 2.91	13.1
Almaken control	0	34.10 ± 1.17	21.10 ± 2.40	0
Almaken mutant lines	100	48.81 ± 4.87	35.73 ± 1.34	69.3 **
Almaken mutant lines	200	47.27 ± 6.65	23.96 ± 1.33	13.2
Eritrosperumum control	0	31.10 ± 1.25	11.33 ± 2.20	0
Eritrosperumum mutant lines	100	46.98 ± 8.27	26.92 ± 3.87	137.6 ***
Eritrosperumum mutant lines	200	48.75 ± 5.74	27.14 ± 3.95	139.5 ***

Note: Values are presented as mean ± standard deviation (SD) of three biological replicates (*n* = 3). Percentage increase was calculated relative to the respective control. Asterisks indicate significant differences compared with the corresponding control according to Dunnett’s multiple comparison test (** *p* < 0.01, *** *p* < 0.001).

**Table 2:** Summary of two-way ANOVA results for the effects of genotype, gamma irradiation dose, their interaction, and block on agronomic and nutritional traits.

Trait	Genotype F (df = 2.16)	*p* Value	Dose F (df = 2.16)	*p* Value	Genotype × Dose F (df = 4.16)	*p* Value	Block F	Block *p* Value (df = 2.16)
Biological yield	54.11	7.57 × 10^−8^	304.62	1.84 × 10^−13^	37.15	6.56 × 10^−8^	0.08	0.928
Protein content (%)	1.21	0.323	3.004	0.078	0.36	0.831	2.56	0.109
Fe (mg kg^−1^)	62.71	2.68 × 10^−8^	419.65	1.50 × 10^−14^	58.64	2.35 × 10^−9^	4.36	0.031
Zn (mg kg^−1^)	12.57	5.24 × 10^−4^	169.00	1.74 × 10^−11^	52.71	5.17 × 10^−9^	1.36	0.285
Phytic acid (mg g^−1^)	936.43	2.65 × 10^−17^	149.19	4.50 × 10^−11^	20.80	3.53 ×10^−8^	0.58	0.574
Phy:Fe molar ratio	616.72	7.23 × 10^−16^	174.16	1.38 × 10^−11^	4.82	9.59 × 10^−3^	5.66	0.014
Phy:Zn molar ratio	188.08	7.68 × 10^−12^	209.34	3.37 × 10^−12^	49.61	8.07 × 10^−9^	1.18	0.33

Note: df_1_ and df_2_ denote the numerator (between-groups) and denominator (within-groups/error) degrees of freedom, respectively. For genotype, irradiation dose, and block, df_1_ = 2; for genotype × irradiation dose interaction, df_1_ = 4; residual df_2_ = 16.

## Data Availability

The raw data supporting the conclusions of this article will be made available by the authors on request.
